# ECMO PAL VV: using deep neural networks for survival prognostication in venovenous extracorporeal membrane oxygenation

**DOI:** 10.1186/s13054-026-06032-7

**Published:** 2026-04-18

**Authors:** Andrew F. Stephens, Michael Šeman, Riley Hackwill, Arne Diehl, David Pilcher, Ryan P. Barbaro, Daniel Brodie, Vincent Pellegrino, David M. Kaye, Shaun D. Gregory, Carol L. Hodgson

**Affiliations:** 1 Advanced Cardiorespiratory Engineering Laboratory, Centre for Biomedical Technologies, Queensland University of Technology, Brisbane, Australia; 2 School of Electrical Engineering and Robotics, Queensland University of Technology, Brisbane, Australia; 3 School of Public Health and Preventive Medicine, Monash University, Melbourne, Australia; 4 Department of Cardiology, Western Hospital, Melbourne, Australia; 5 Department of Intensive Care and Hyperbaric Medicine, The Alfred Hospital, Melbourne, Australia; 6 Pediatric Critical Care Medicine, Susan B. Meister Child Health Evaluation and Research Center, University of Michigan, Ann Arbor, MI USA; 7 Division of Pulmonary and Critical Care Medicine, School of Medicine, Johns Hopkins University, Baltimore, MD USA; 8 School of Mechanical, Medical, and Process Engineering, Queensland University of Technology, Brisbane, Australia; 9 Department of Cardiology, The Alfred Hospital, Melbourne, Australia; 10 Australian and New Zealand Intensive Care Research Centre, Melbourne, Australia

**Keywords:** Artificial intelligence, ARDS, Prognostication, Risk adjustment, Survival score, ECMO

## Abstract

**Background:**

Prognostication for venovenous extracorporeal membrane oxygenation (ECMO) outcomes is crucial for risk-adjusting centre performance. This study aimed to leverage a large, multicentre, international database to develop and evaluate AI-driven models for predicting survival to hospital discharge of adult patients receiving venovenous ECMO. The model was called ECMO PAL VV (ECMO – Predictive Algorithm for VV).

**Methods:**

Training and temporal validation data were sourced from the Extracorporeal Life Support Organization Registry (ELSO), 39,501 patients across 660 hospitals. Deep neural networks were trained on all adult patients receiving VV ECMO between 2017 and 2023 (*N* = 35,182) to predict survival to hospital discharge. Temporal validation was performed on registry data cases from 2024 (*N* = 4,318). Model predictions were compared against published venovenous ECMO outcomes scores using the validation cohort.

**Results:**

Internal training yielded an accuracy of 79% and an area under the receiver operating characteristic curve (AUC) of 0.87. Temporal validation revealed a drop in accuracy to 73% with an AUC of 0.78, primarily due to a reduction in sensitivity to mortality prediction (71% to 57%). ECMO PAL VV outperformed published venovenous ECMO scores, which had accuracies of 65% (RESP) and 60% (Lazzeri score) for predictions on the validation data.

**Conclusions:**

ECMO PAL VV demonstrated strong accuracy on contemporary international registry data (73%) with strong sensitivity (81%) and precision (77%) to predict survival to hospital discharge, outperforming existing published scores. ECMO PAL VV has the potential to improve risk adjustment and enable data-driven healthcare.

**Graphical abstract:**

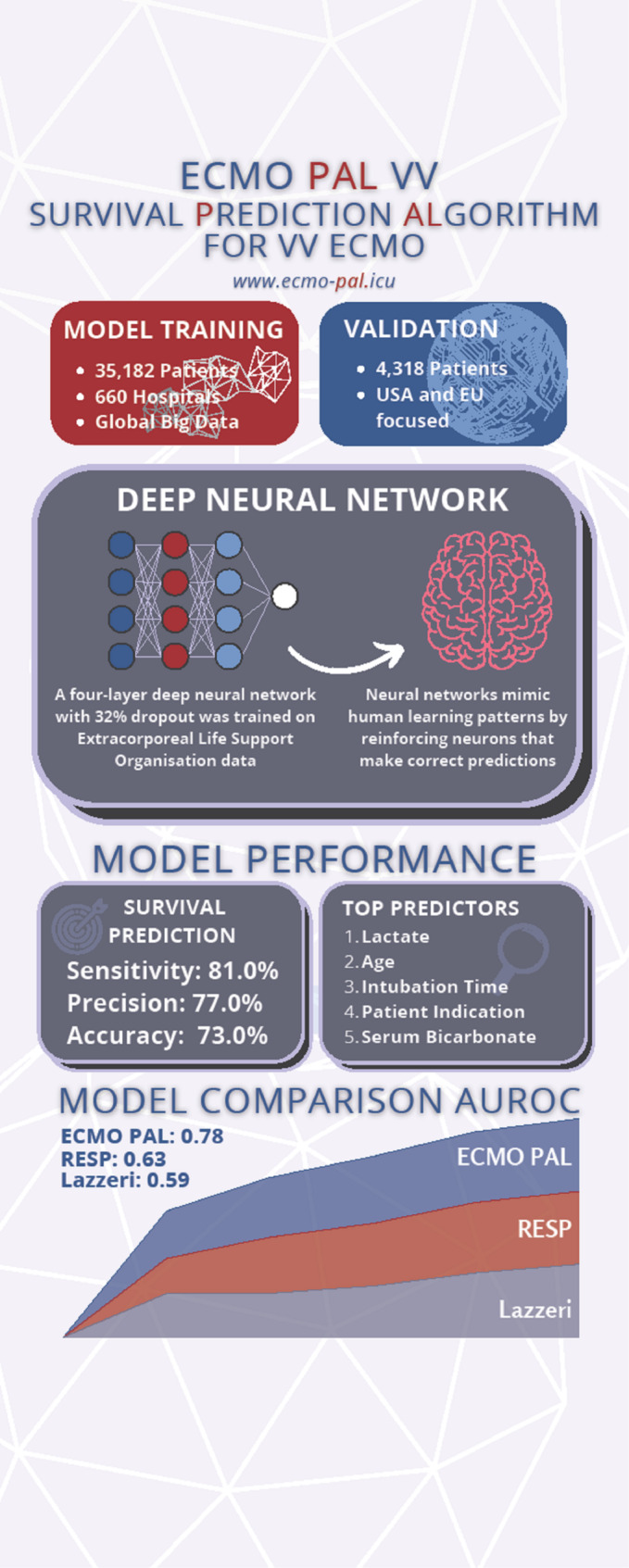

**Supplementary Information:**

The online version contains supplementary material available at 10.1186/s13054-026-06032-7.

## Introduction

Venovenous extracorporeal membrane oxygenation (VV ECMO) is a complex therapy that requires a high level of expertise in therapeutic decision-making and prognostication. Medical management influences patient survival and long-term outcomes [1]. Factors such as disease aetiology, patient comorbidities, demographics, disease progression, and long-term outcomes must all be considered prior to ECMO initiation and during patient treatment [1, 2]. Medical prognostication tools have found widespread clinical, administrative, and research use and can help facilitate data-driven approaches around goals of care, hospital benchmarking across regions, and track hospital performance over time [2, 3]. Previously, ECMO patient survival indicators have been developed based on traditional statistical modelling methods, such as logistic regression, which may be limited in their capacity to describe the complex feature interactions that drive ECMO outcomes [2].

Artificial intelligence (AI) has begun to proliferate across medicine, with substantial potential to capture complex interactions and patterns that traditional statistical methods otherwise overlook. Several AI models for VV ECMO prognostication have been previously developed for predicting neurological outcomes, decannulation success, and patient mortality [4–7]. These previously developed models have focused on specific disease aetiologies or have been derived from a single centre or region, limiting global generalisability.

This study aimed to leverage a large, multicentre, international database to develop and evaluate an AI-driven model (called ECMO PAL VV – ECMO Predictive Algorithm for VV) for predicting survival to hospital discharge of patients supported by VV ECMO. The secondary aim was to compare the developed model against existing published scores. AI-powered prognostication tools can be used by clinicians and administrators to improve ECMO centre quality control, benchmarking and resource allocation.

## Methods

These methods outline the full study protocol and adhere to the Transparent Reporting of a Multivariable Prediction Model for Individual Prognosis or Diagnosis with Artificial Intelligence (TRIPOD-AI) and Minimum Information for Medical AI Reporting (MINIMAR) guidelines [8, 9].

### Data and inclusion

Data were obtained retrospectively from patients enrolled in the Extracorporeal Life Support Organisation (ELSO) registry (elso.org). The ELSO Registry collects data on the admission, initiation, maintenance, complications, and outcomes of ECMO patients worldwide. ELSO data is collected from over 660 ECMO centres globally, primarily in the intensive care setting, operating theatre and emergency department. The patient cohort included all adult patients (≥ 18 years) who received VV ECMO between January 15, 2017, and December 31, 2023. This epoch was chosen to coincide with the latest major update to the ELSO registry reporting variables (January 2017). There were 37,665 registry patients present during this epoch. Patients with mixed cannulation modes, such as venovenous-arterial ECMO, were not included.

**Fig. 1:**
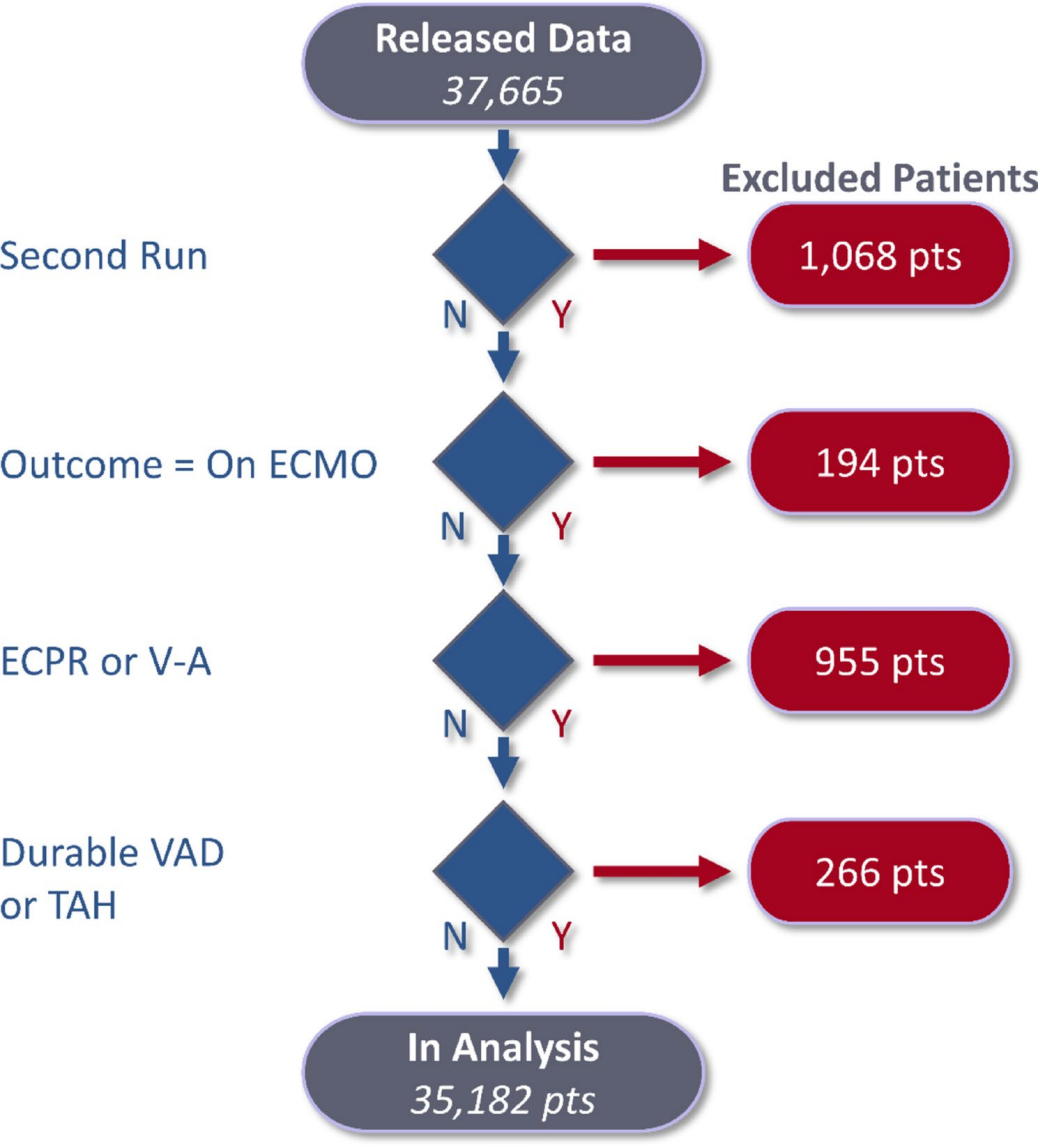
Flow chart of the exclusion process. ECPR – Extracorporeal membrane oxygenation cardiopulmonary resuscitation; TAH – Total artificial heart; V-A – Venoarterial extracorporeal membrane oxygenation; VAD – Ventricular assist device

In cases where patients had multiple ECMO runs or a prior history of ECMO, only the first ECMO run was included. Four exclusion criteria were defined in consultation with a multidisciplinary group of ECMO experts; (1) patients on their second or higher ECMO run; (2) patients indicated as *still on ECMO* whose outcome was unknown at the time of reporting (predominantly transferred to a non-ELSO participating centre and lost to follow-up); (3) patients who were present in the VV registry but listed as being ECPR or found to have only a venous drainage cannula and arterial return cannula (incorrectly classified as VV or mode converted after initiation), or whose primary diagnosis was *cardiogenic shock*; (4) presence of a durable ventricular assist device or total artificial heart. Following exclusion, 35,182 patients remained for analysis (Fig. 1).

### Data pre-processing

All pre-ECMO registry variables were considered for analysis. Pre-ECMO haemodynamics and blood gases were defined as the closest reading before ECMO initiation, collected no more than 6 h before ECMO initiation [10]. Pre-ECMO support included procedures or interventions occurring up to 24 h before ECMO initiation [10]. Data pre-processing consisted of deterministic manual data cleaning, data clustering, imputation, and feature scaling. Manual cleaning involved grouping ICD-10 diagnosis codes into broader indications, as defined in the literature by other scores such as the Charlson Comorbidity Index, Classification of Hospital-Acquired Diagnoses (CHADx), Multipurpose Australian Comorbidity Scoring System (MACCS), and EXCEL registry data definitions [11–15]. Secondary ICD-10 code clustering was performed independently by two clinicians (MS and AD) and finalised through negotiation. Furthermore, clustering of Current Procedural Terminology (CPT) codes was undertaken to identify pre-ECMO cardiothoracic surgery and other major pre-ECMO surgeries [16].

**Fig. 2:**
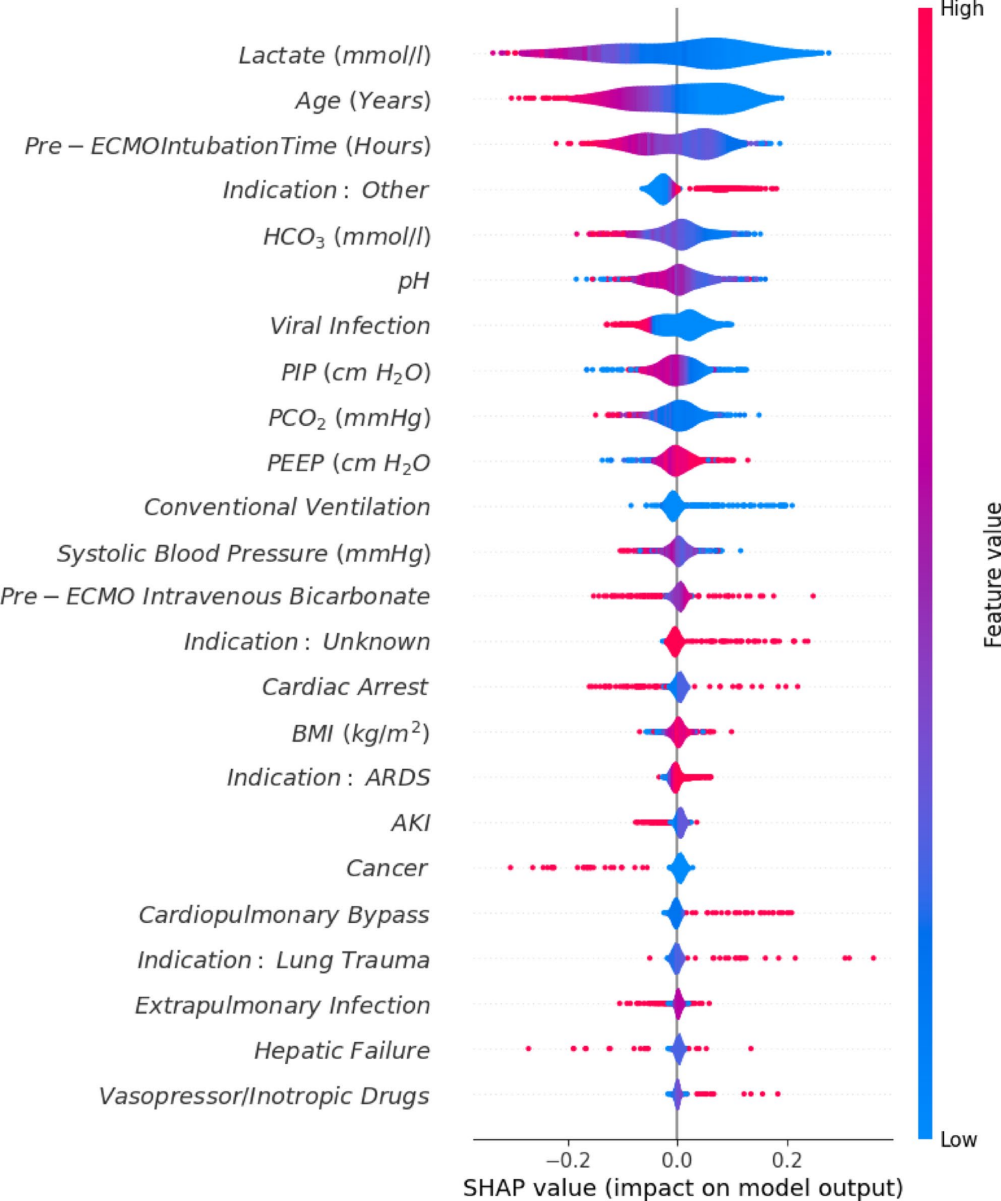
Shapley Additive Explainer (SHAP) values indicate the influence of each variable on the model’s output. The colour of each dot represents the value of an individual patient data point, with pink being a high variable value and blue being a low variable value. The arm length represents that data point’s contribution to a patient-specific outcome in combination with all other variables for that patient. The thickness of the violin shows the distribution of patients with that value. Variables are ranked in descending order of predictive importance

In cases where the hospital admission diagnosis was given instead of the VV ECMO indication, a deterministic method was used to derive the ECMO indication, combining additional patient data such as surgical procedures and pre-ECMO support (supplemental online material - Fig. 1). VV ECMO indications were clustered into categories of acute respiratory distress syndrome, asthma, coronavirus disease-19 (COVID-19), chronic end-stage lung diseases (CESLD), focal lung disease (including pneumonias), lung transplant, lung trauma, pulmonary vasculitis and haemorrhage, toxin, other (comprising any primary ICD-10 code that was non-cardiac or -respiratory), and unknown (supplemental online materials – Fig. 2).

Continuous data were checked for outliers and probable erroneous entries by hard data limits as agreed by clinical consensus (supplemental online materials – Table 1). Data outside these limits were truncated or removed for later imputation, as indicated in the table. Variables with more than 50% missingness were removed entirely from the analysis [17]. All data were min-max scaled, and missing data were imputed using *stochastic iterative imputations by chained equations* to a tolerance of 0.005. Training and validation data were imputed separately and included the outcome variable. A total of 85 variables were included in the modelling process. A comprehensive feature list with missingness is available in the supplemental materials (Table 2).

### Machine learning methods

Based on previous experience, a neural network architecture was used for predicting survival to hospital discharge as the primary endpoint [18]. All 85 variables were included in the initial model, which underwent hyperparameter tuning via Tree-Structured Parzen Estimators (a Bayesian optimisation algorithm) [19]. Following optimisation, feature selection was performed to limit model overfitting and improve model deployability. Features with low predictive value were removed piecewise using permutation feature importance, retaining only variables with predictive power greater than 0.005. For every ten features removed in this fashion, hyperparameter tuning was performed to refit the model, ensuring consistent performance. Pearson correlation coefficients were used to assess collinearity among the remaining features, and the feature with the least predictive value was removed if its removal did not affect ECMO PAL VV performance (accuracy reduction of less than 1%). ECMO PAL VV outputs a continuous percentage likelihood of survival to hospital discharge, with values ≥ 50% binarised to a prediction of survival for metric analysis (a prediction of ≥ 50% means survival; Supplemental online materials - Table 3).

### Model evaluation

ECMO PAL VV training and assessment of fit were performed under five-fold cross-validation. The key values of interest were sensitivity (ratio of correct predictions [survival or mortality] to observed outcome), precision (positive predictive value – ratio of correct predictions to total predictions), overall model accuracy (ratio of correct predictions to incorrect predictions), and area under the receiver operating characteristic (AUC). Shapley Additive Explanations (SHAP) were used to understand feature importance and illustrate each feature’s effect on model predictions [20]. Partial dependence plots explored variable interactions in more detail [21, 22].

Validation was performed on a new epoch of data from the ELSO database, comprising all adult VV ECMO patients in 2024 (*N* = 4,318), to assess the model’s performance in contemporary patients. The validation data were cleaned, imputed, and scaled in the same manner as the training data, and predictions were made using the same metrics as during training.

A sensitivity analysis was performed to investigate model robustness to missing variables. Up to ten variables for each patient from the ELSO validation data were replaced with the median value from the training data, simulating a default value being used in the prediction tool. Predictions were made on each patient, and the overall accuracy was plotted against the number of median imputed variables. A calibration belt was generated on the training data by evaluating the upper and lower 95% confidence intervals of the model at each outcome probability decile (0 to 100% survival prediction).

Finally, ECMO PAL VV predictions were compared against previously published ECMO survival scores, RESP, and the Lazzeri score, based on calculability using ELSO registry data [23, 24]. Each of the evaluated scores made predictions on the temporal validation data, and the AUCs and overall accuracy of each score were compared against the developed model’s prediction scores.

## Results

### Training patient demographics and clinical data

The mean patient age was 48 ± 11 years, with 64% male and 36% female (Table 1). Patient races were recorded as Asian (10.6%), Black (11.0%), Hispanic (12.3%), Multiple (5.7%), White (49.9%), other (6.4%), and unknown (4.1%). Training data came from 633 participating centres internationally. No socioeconomic data were available. 34% of patients had COVID-19 as either a primary or secondary diagnosis. The distribution of survival outcomes can be found in the online supplemental materials (Table 4).

### Feature importance and effects on output

Permutation feature importance and Pearson correlation were used to identify and reduce the model input variables. Peak inspiration pressure (PIP) and positive end-expiratory pressure (PEEP) were found to be modestly correlated (+ 0.5), as were PCO_2_ and pH (-0.6), and an indication of *focal lung disease* and risk factor of *viral infection* (0.7). Piecewise removal of highly correlated variables was conducted, resulting in only the removal of *focal lung disease*, with an acceptable corresponding 0.5% reduction in overall accuracy. Through feature selection, 85 features were reduced to 24 features with predictive power > 0.005. Feature importance was explored post-hoc using SHAP explainers (Fig. 2).

The variables with the highest predictive value were lactate, age, pre-ECMO endotracheal intubation time, an ECMO indication of *other*, and pre-ECMO serum bicarbonate (HCO_3_). Many variables had a clear monotonic relationship to the outcome. For example, having a higher lactate, being intubated for longer before ECMO commencement, and being older were all monotonically associated with lower survival (Fig. 2). Conversely, patients with an indication of *other*, having a higher PEEP, and those who received pre-ECMO cardiopulmonary bypass were associated with better outcomes. Reported sex was not found to contribute to outcomes.

Variables with complex effects on patient outcomes included pH, PIP, and extrapulmonary inflammation/infection, reflecting a strong interaction between variables and highlighting possible prior patient selection biases. Interactions between lactate, pH, and HCO3 were explored using partial dependence plots, showing an area of high survival with lactate levels between 3.8 and 9.5 mmol/L and HCO3 levels below 24.6 mmol/L. Another area of high survival was demonstrated, with lactate levels between 3.0 and 10.1 mmol/L and pH levels between 7.0 and 7.2 (supplemental online materials, Fig. 3).

**Table 1:** Baseline characteristics of extracorporeal membrane oxygenation (ECMO) patients included in the training data prior to imputation

Patient data	All patients median [IQR]	Survived median [IQR]	Non-survivors median [IQR]
Age (Years)	48.3 [36.2–58.7]	45.3 [33.7–56.2]	52.7 [41.4–61.2]
Body Mass Index (kg/m^2^)	29.9 [25.4–35.9]	30.0 [25.4–36.2]	29.9 [25.5–35.4]
Lactate (mmol/L)	1.8 [1.2–3.2]	1.7 [1.1–2.9]	2 [1.3–3.7]
pH	7.3 [7.2–7.4]	7.3 [7.2–7.4]	7.3 [7.2–7.3]
HCO_3_ (mmol/L)	26 [22–31]	26 [22–31]	26 [22–32]
PIP (cmH_2_O)	32 [27–37]	32 [26–37]	33 [23–38]
PEEP (cmH_2_O)	10 [12–15]	12 [10–15]	12 [10–15]
PaO_2_ (mmHg)	67 [56–84]	68 – [65–86]	66 [55–82]
PaCO_2_ (mmHg)	59 [48–75]	58 [47–73]	61 [50–77]
Systolic Blood Pressure (mmHg)	113 [99–130]	114 [100–131]	112 [98–128]
Pre-ECMO Intubation Time (hours)	36 [9–117]	27 [8–100]	55 [13–134]
Reported Sex (Male)	64.4%	62.4%	67.0%

**ECMO Indication**	**Proportion of** ***N*** ** = 35,182**	**Proportion of** ***N*** ** = 20,915**	**Proportion of** ***N*** ** = 14,267**
ARDS	9,549 (27.1%)	5,608 (26.8%)	3,941 (27.6%)
Asthma	839 (2.4%)	763 (3.6%)	76 (0.5%)
Chronic End-Stage Lung Disease	1057 (3.0%)	705 (23.9%)	352 (2.5%)
Coronavirus Disease-19	10,072 (28.6%)	4,995 (23.9%)	5,077 (35.6%)
Focal Lung Disease*	4,128 (11.7%)	2,606 (12.5%)	1,522 (10.7%)
Lung Transplant Failure	2,382 (6.8%)	1,553 (7.4%)	829 (5.8%)
Lung Trauma	1,158 (3.3%)	800 (3.8%)	355 (2.5%)
Toxins	45 (0.1%)	29 (0.1%)	16 (0.1%)
Pulmonary Vasculitis and Haemorrhage	146 (0.4%)	89 (0.4%)	57 (0.4%)
Other	3,449 (9.8%)	2,595 (12.4%)	1,387 (9.7%)
Unknown	1,830 (5.2%)	1,175 (5.6%)	655 (4.6%)

**Pre-ECMO Status**	**Proportion of** ***N*** ** = 35,182**	**Proportion of** ***N*** ** = 20,915**	**Proportion of** ***N*** ** = 14,267**
Extrapulmonary Infection or Inflammation	7,975 (22.6%)	4,174 (20.0%)	3801 (26.6%)
Acute Kidney Injury	6,166 (17.5%)	3,123 (14.9%)	2043 (21.3)
Cardiac Arrest	3,631(10.3%)	1,855 (8.9%)	1776 (12.4%)
Cancer	819 (3.2%)	490 (2.3%)	645 (4.5%)
Cardiopulmonary Bypass	962 (2.7%)	665 (3.2%)	297 (2.1%)

**Outcomes**	**Mean [IQR]**	**Mean [IQR]**	**Mean [IQR]**
ECMO Support Duration (Hours)	252 [119–525]	217 [117–436]	324 [127–637]
Survival to Hospital Discharge	59.4%	100.0%	0.0%

Patient data, blood gases, and haemodynamics were collected up to 6 h before ECMO initiation, while pre-ECMO statuses occurred within 24 h before ECMO initiation

*Includes non-COVID-19 pneumonias. ARDS – Acute Respiratory Distress Syndrome; ECMO – extracorporeal membrane oxygenation; IQR – interquartile range; N – number of entries

For instance, a high lactate (bright pink colour) was strongly associated with mortality outcomes (dots plotted to the far left). As lactate decreases (colour changes from pink to blue), it becomes associated with survival (dots to the right). The further left or right the dot is plotted, the more strongly it is associated with a survival or mortality.

### Internal model performance and model validation

Internal five-fold cross-validation revealed an accuracy of 78.7 ± 0.5% and an AUC of 0.868 ± 0.001 (Table 2). Temporal validation of new patients from the ELSO registry yielded an overall accuracy of 73.0% with an AUC of 0.780. The drop in AUC was mainly caused by a reduction in in-hospital mortality sensitivity between the internal training and temporal validation (70.7% vs. 57%0.0, respectively) (Table 2).

**Table 2:** ECMO PAL VV metrics from internal five-fold cross-validation (*N* = 35,182) and temporal validation (*N* = 4,318)

Metric	Internal evaluation	Temporal validation	Validation 99% CI
Survival to discharge sensitivity (%)	84.6 ± 0.6	81.0	[79.0–82.9]
Survival to discharge precision (%)	79.7 ± 0.5	77.0	[74.9–79.0]
Hospital mortality sensitivity (%)	70.7 ± 1.0	57.0	[53.7–60.3]
Hospital mortality precision (%)	77.3 ± 0.7	63.0	[59.8–66.2]
Overall accuracy (%)	78.7 ± 0.5	73.0	[71.2–74.8]
AUC	0.868 ± 0.001	0.780	-

*AUC – area under the receiver operating characteristic curve; CI – confidence interval; N – number of entries. Confidence intervals reflect uncertainty due to sample size and were calculated using the Exact Binomial method

Sensitivity analysis revealed a decrease in accuracy from 71% to 60% as the number of randomly missing variables increased, up to a maximum of ten (supplemental online materials – Fig. 4). Meanwhile, the calibration belt showed a slight underprediction of survival in patients with poor predicted outcomes (0.1–0.4) and a modest overprediction in patients with strong predicted outcomes (0.8–0.9). The calibration mean absolute error was 0.07, and the sum of squared errors was 0.13 (supplemental online materials Fig. 5). A histogram of ECMO PAL VV predictions on the global data is given in supplemental online materials Fig. 6).

**Fig. 3:**
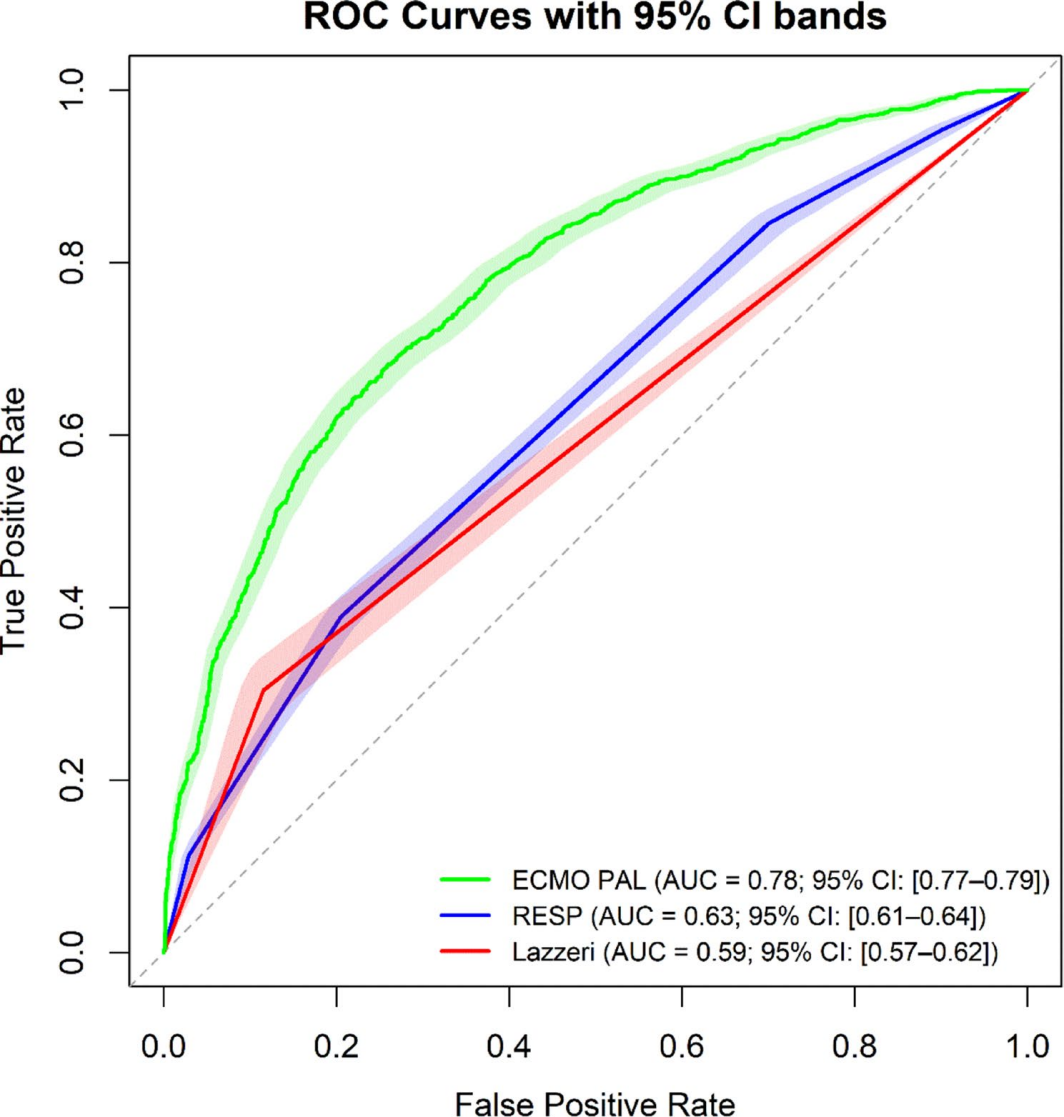
Area under the receiver operating characteristic curve (AUC) for ECMO PAL VV, the Lazzeri Score, and the RESP score. The dotted grey line represents an AUC of 0.5 (prediction is completely random)

ECMO PAL VV outperformed the RESP and Lazzeri scores on the ELSO temporal validation dataset. ECMO PAL VV and RESP made predictions for all patients (*N* = 4,318), whereas the Lazzeri score predicted only patients with ARDS, in line with its use-case recommendations (*N* = 1,299). ECMO PAL performed best, with an accuracy of 73% and AUC of 0.78, followed by RESP with an accuracy of 65% and AUC of 0.63, and the Lazzeri score with an accuracy of 60% and AUC of 0.59 (Fig. 3).

## Discussion

### Key findings

This study developed an AI prediction algorithm for VV ECMO patient survival using a large, international dataset (ECMO PAL VV). The model was found to be robust to temporal validation and demonstrated strong sensitivity (81.0%) and precision (77.0%) in predicting hospital survival. The model demonstrated an overall accuracy of 73.0% in a temporal validation of patients receiving ECMO included in the ELSO registry but had lower sensitivity when predicting mortality (57.0%). The model identified several key drivers of survival in VV ECMO patients.

Consistent with previously published AI models for venoarterial ECMO prognostication, the developed model demonstrated that the key drivers of outcome were pre-ECMO lactate, age, pre-ECMO intubation time, and serum bicarbonate, with underlying aetiology playing a less critical role in the model’s prognostic process [18]. This highlights the significant difference between clinical thinking and machine thinking, which incorporates patient disease aetiology into the prognosis by examining blood gas and haemodynamic factors without the need for disease labelling or phenotyping. Although the top model drivers were primarily associated with mortality, the developed model demonstrated higher sensitivity and precision to the primary outcome (survival to hospital discharge), compared to other published risk prediction models for VV ECMO.

Several previous studies have used AI to predict VV ECMO patient outcomes or complications [4, 5, 7]. Most notably, a large ELSO registry study was used to train and predict acute brain injury in 37,473 patients across 676 centres, with an AUC of 0.70 on an internal leave-one-out cross-validation [5]. Another, smaller study used tree-based machine learning to train and predict neurological outcomes in 99 VV ECMO patients from a single centre, achieving an internal leave-one-out cross-validation of 0.87 AUC [4]. Researchers have also used machine learning models to train and predict 90-day mortality in 368 VV ECMO patients across 16 Korean centres (external validation AUC = 0.75) [7]. However, many of these studies are limited by their reliance on single-centre data or a lack of robust validation methods. Internal validation techniques, such as leave-one-out cross-validation, primarily assess model fit and do not reliably indicate generalisability to future or external populations.

The most predictive variable was lactate, with a high missingness rate of 48.8%. Nonetheless, lactate was included in the model due to its known clinical importance, as described in multiple other ECMO prognostication scores [5, 18, 25, 26]. Despite the number of missing values, lactate imputation confidence was high because of the large sample sizes of available lactate data (> 15,000 for training and > 2,000 for validation), which allowed a representative distribution to be created for imputations. Robust, stochastic imputation methods can generate complete, accurate synthetic datasets when supplied with appropriate variance and sample size [27, 28].

This study used an international registry for training and validation. However, it should be noted that the data within the registry is primarily from North America (68.2%) and Europe (17.2%). Data from Asia Pacific comprised 6.2%, while the remaining patients were from South and West Asia and Latin America. Given the highly data-driven nature of AI models, it is expected that model performance will be lower when used in regions with fewer data within the training cohort. Model accuracy across all regions will continue to improve as new data is collected by the ELSO registry and models are updated with time. In the meantime, caution should be used when interpreting model outputs from regions outside Europe and North America.

### Future directions

ECMO registry data were used to develop ECMO PAL VV, introducing selection bias, as the decision to initiate ECMO had already been made by the attending clinician (including their internal decision-making processes and assessment of the likelihood of survival). Patients with an expected mortality risk perceived as too high by their attending clinician did not receive ECMO and, thus, were not entered in this registry. Due to this survival bias, ECMO PAL VV is currently unsuitable for decision-making about goals of care for individual ECMO patients without prospective validation. However, ECMO PAL VV has immediate utility in risk adjustment. Globally, ECMO is frequently delivered in a hub and spoke model, with a large, well-equipped, central centre accepting referrals from smaller peripheral centres that perform initiation and maintenance of “simpler” cases [29]. Risk adjustment of ECMO patient outcomes is critical to ensure that centres treating significantly comorbid or high-risk patients are not perceived as underperforming compared to peripheral centres.

ECMO PAL VV and ECMO PAL VA (respiratory and cardiac) are currently being integrated into ELSO reporting systems as risk-adjustment tools for benchmarking centre performance. This will allow over 660 member hospitals to access generalised risk-adjusted outcome performance at a regional, state, or national level, or compare with other centres of a similar size globally. Although the current model has lower mortality sensitivity, its strong survival-prediction capability can be leveraged for risk adjustment based on survival. This would mean identifying patients who should have survived and pinpointing where centres are falling short, rather than the classical mortality-based risk-adjustment approach. ESLO registry integration represents the first global deployment of an AI-powered application for ECMO.

### Strengths and limitations

ECMO PAL VV was trained on a large international registry dataset comprising data from 633 hospitals, thereby greatly improving its global generalisability compared with other published models. ECMO PAL VV was validated using temporal data from 660 ECMO centres to ensure its contemporary utility and compared to existing models. True external validation was not possible in this study as the author’s institutions all contribute directly to the ELSO registry, resulting in overlap with the temporal validation cohort. Robustness of validation should be at the forefront of criticism when assessing proposed AI models for medical applications. To ensure high quality, published AI models must undergo temporal or external validation and demonstrate consistent performance across diverse regions, unless specifically designed for localised use.

This study had several limitations. Firstly, this study used a dataset that was significantly affected by COVID-19 (the 2017–2023 training dataset). The global mortality rate for VV ECMO rose by 5% during COVID (2020 to 2023), undoubtedly contributing to the reduction in mortality sensitivity and precision observed in the temporal validation subset (2024). Among the included training patients, 33.4% had COVID-19 as their primary indication for ECMO. As such, COVID-19 consistently ranked among the top 10 variables with predictive power across early models. In contrast, only 2.2% of patients in the temporal validation dataset had COVID-19, reflecting the sharp decline in ECMO use for COVID-19 in recent years. A sensitivity analysis was conducted to determine the effect of COVID-19 as a variable in the temporal validation dataset, and a further decrease in mortality sensitivity was observed (57% to 52%) (Supplemental Online Materials - Table 4). Due to the low contemporary relevance, a clinical decision was made to remove COVID-19 as a variable in the final model. This was achieved by removing COVID-19 as an independent indication for ECMO and recategorizing those patients with other types of pneumonia under *focal lung disease*.

Another limitation was the varying quality of available registry data, with some centres consistently entering higher-quality data than others. Experience with the ELSO registry has shown an overall trend toward higher-quality data entry as the registry matures. As the data quality improves and more data is added, these AI models will improve commensurately. Newer models should also focus on more meaningful, longer-term outcomes beyond hospital mortality that are not currently captured in the ELSO registry.

Although the model has demonstrated good accuracy on patient registry data, it captures data at only a single time point at ECMO initiation. As such, the clinical interventions are not captured, nor are the resulting changes in the patient trajectory. This means that model accuracy has an upper limit, making it difficult to use for predicting prospective outcomes at the individual patient level. Models that incorporate additional time points could capture patient trajectories throughout their support duration and provide more accurate forecasts of patient weaning readiness and outcomes.

## Conclusions

This study aimed to leverage a large, multicentre, international database to create AI-driven models for predicting survival to hospital discharge of VV ECMO. ECMO PAL VV demonstrated good accuracy and AUC, but lower sensitivity and precision in predicting in-hospital mortality. ECMO PAL VV outperformed published scores in a large patient cohort of 4,318 patients. ECMO PAL is currently being rolled out as part of ELSO risk-adjustment reporting for global centre benchmarking. ECMO PAL can be continuously improved as new data are added to the ELSO registry. In the future, ECMO PAL can be expanded to include additional time points to provide more accurate, contemporaneous predictions and advance the model as a tool for data-driven clinical decision-making.

## Supplementary Information

Below is the link to the electronic supplementary material.

Supplementary Material 1.

## Data Availability

This article’s data cannot be shared publicly as it is a private health registry contributed to by participating hospitals through the Extracorporeal Life Support Organization. Data requests can be addressed directly to ELSO (elso.org). You can try ECMO PAL VV at [ecmo-pal.icu] . The source code is available upon request.
