# Supplementary material for: ECMO PAL VV: using deep neural networks for survival prognostication in venovenous extracorporeal membrane oxygenation
Source: Crit Care. 2026 Apr 18;30:279. doi: 10.1186/s13054-026-06032-7 (PMC13224564; doi:10.1186/s13054-026-06032-7)
Supplement: Supplementary file 1 — Supplementary Material 1. [file 13054_2026_6032_MOESM1_ESM.docx]

**Supplemental Electronic Materials:** **ECMO PAL V-V: Using Deep Neural Networks for Survival Prognostication in Venovenous Extracorporeal Membrane Oxygenation**

**Contents**

[Supplemental Figure 1 2](#_Toc221006957)

[Supplemental Figure 2 3](#_Toc221006958)

[Supplemental Table 1 4](#_Toc221006959)

[Supplemental Table 2 5](#_Toc221006960)

[Supplemental Table 3 7](#_Toc221006961)

[Supplemental Table 4 8](#_Toc221006962)

[Supplemental Figure 3 8](#_Toc221006963)

[Supplemental Figure 4 9](#_Toc221006964)

[Supplemental Figure 5 10](#_Toc221006965)

[Supplemental Figure 6 11](#_Toc221006966)

[Supplemental Table 4 11](#_Toc221006967)

## Supplemental Figure 1


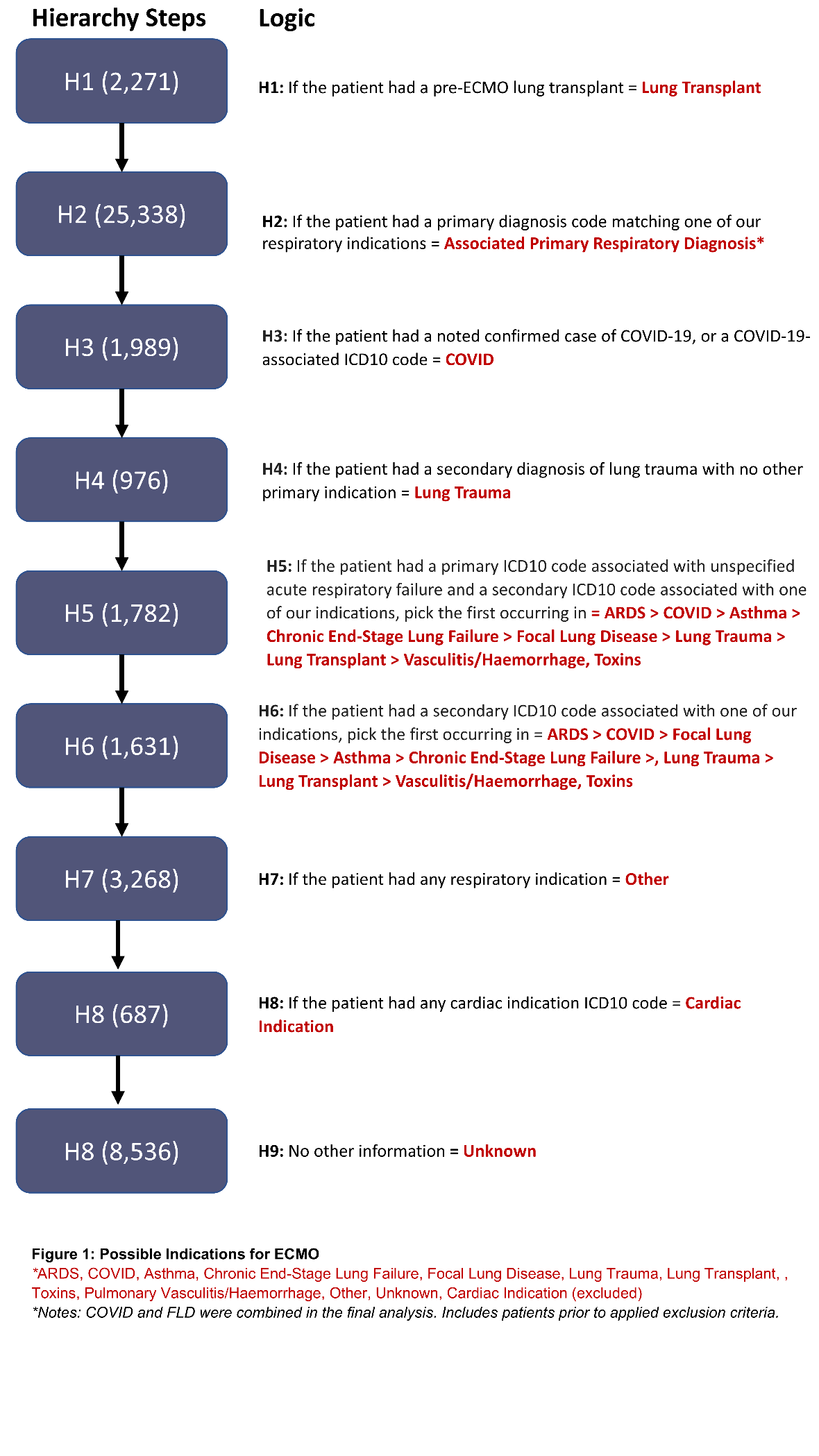


## Supplemental Figure 2


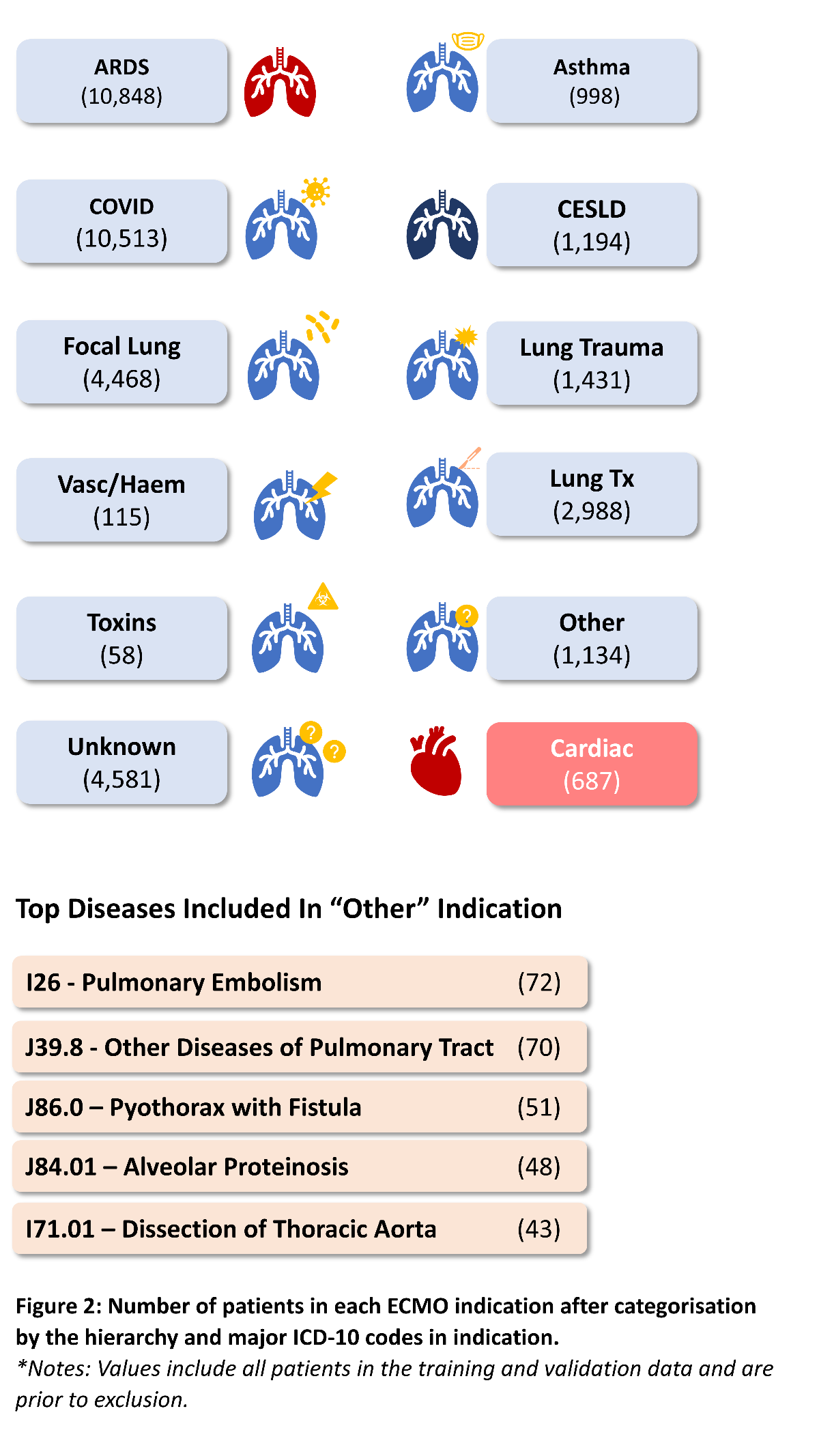


| Supplemental Table 1 Hard limits for continuous variables. | |
| --- | --- |
|  |  |
| Feature | Exclusion Criteria |
| Weight^†^ | < 20 kg |
| Height^†^ | < 100 cm OR (> 100 & < 135 cm if BMI > 90) |
| BMI^††^ | > 75 |
| PaCO_2_^‡^ | < 15, > 250 mmHg |
| PaO_2_^‡^ | < 10, > 650 mmHg |
| HCO_3_^†^ | < 1 |
| SaO_2_^‡^ | < 10 |
| Pre-ECMO Intubation Time ^‡^ | > 605 hours* |
| FIO_2_^‡^ | < 21% |
| Lactate^†^ | > 20 |
| SBP^†^ | < 250 |

* based on the 95^th^ percentile of available data.

^†^Value imputed

^††^Value calculated from height and weight

^‡^Value truncated

## Supplemental Table 2

Full list of variables included in the study, along with their initial missingness. For access to the ICD-10 codes and CPT codes present in the data please contact [research.andrew.stephens@gmail.com](mailto:research.andrew.stephens@gmail.com) directly.

| Variable Name | Variable Type | Missing (%) | Minimum Value | Maximum Value |
| --- | --- | --- | --- | --- |
| Demographics | | | | |
| Age (years) | Continuous | 0 | 18 | 81 |
| Male | Boolean | 0.3 | 0 | 1 |
| Height (cm) | Continuous | 8.8 | 100 | 231 |
| Weight (kg) | Continuous | 1.4 | 25 | 458 |
| Year of ECMO | Discrete | 0 | 2017 | 2023 |
| Pre-ECMO Haemodynamics and Blood Gasses | | | | |
| SBP (mmHg) | Continuous | 22.2 | 10 | 250 |
| DBP (mmHg) | Continuous | 22.3 | 2 | 220 |
| MeanBP (mmHg) | Continuous | 33.7 | 0 | 221 |
| HCO3 | Continuous | 22.2 | 10 | 70 |
| Lactate | Continuous | 48.6 | 0.1 | 20 |
| pH | Continuous | 18.9 | 6 | 8 |
| PaCO2 (mmHg) | Continuous | 22.9 | 15 | 250 |
| PaO2 (mmHg) | Continuous | 23.0 | 10 | 650 |
| SaO2 (%) | Continuous | 34.9 | 10 | 100 |
| Pre-ECMO Support | | | | |
| Ventilator Type | Categorical | 8.2 | 0 | 0 |
| PIP (cmH2O) | Continuous | 36.7 | 0 | 100 |
| PEEP (cmH2O) | Continuous | 27.9 | 0 | 40 |
| FiO2 (%) | Continuous | 24.7 | 21 | 100 |
| Respiratory Rate (Breaths/min) | Continuous | 28.1 | 0 | 100 |
| Hand Bagging | Boolean | 0 | 0 | 1 |
| Log of IntubationTime (hours) | Continuous | 15.5 | -1 | 6.4 |
| Beta-blocker | Boolean | 0 | 0 | 1 |
| Bicarbonate (Intravenous) | Boolean | 0 | 0 | 1 |
| Inhaled anesthetic | Boolean | 0 | 0 | 1 |
| Levosimendan | Boolean | 0 | 0 | 1 |
| Milrinone | Boolean | 0 | 0 | 1 |
| Pulmonary Vasodilator | Boolean | 0 | 0 | 1 |
| Systemic Steroids | Boolean | 0 | 0 | 1 |
| Vasodilator Drugs | Boolean | 0 | 0 | 1 |
| Vasopressor/Inotropic Drugs | Boolean | 0 | 0 | 1 |
| Cardiopulmonary Bypass | Boolean | 0 | 0 | 1 |
| Intraaortic Balloon Pump | Boolean | 0 | 0 | 1 |
| Percutaneous Ventricular Assist Device | Boolean | 0 | 0 | 1 |
| Pacer/ICD | Boolean | 0 | 0 | 1 |
| Renal Replacement Therapy | Boolean | 0 | 0 | 1 |
| THAM - Acidosis correction | Boolean | 0 | 0 | 1 |
| Therapeutic hypothermia < 35 °C | Boolean | 0 | 0 | 1 |
| Indications | | | | |
| ARDS | Boolean | 0 | 0 | 1 |
| Asthma | Boolean | 0 | 0 | 1 |
| COVID-19 | Boolean | 0 | 0 | 1 |
| CESLD | Boolean | 0 | 0 | 1 |
| Focal Lung Disease | Boolean | 0 | 0 | 1 |
| Lung Transplant | Boolean | 0 | 0 | 1 |
| Lung Trauma | Boolean | 0 | 0 | 1 |
| Pulmonary Vasculitis/ Haemorrhage | Boolean | 0 | 0 | 1 |
| Toxins | Boolean | 0 | 0 | 1 |
| Other | Boolean | 0 | 0 | 1 |
| Comorbidities | | | | |
| Acute Pancreatitis | Boolean | 0 | 0 | 1 |
| Acute Thromboembolic Disease | Boolean | 0 | 0 | 1 |
| Acute Kidney Injury | Boolean | 0 | 0 | 1 |
| Anemia (Chronic/Nutritional) | Boolean | 0 | 0 | 1 |
| Antithrombotic / Antiplatelet Thearapy | Boolean | 0 | 0 | 1 |
| Arrhythmia | Boolean | 0 | 0 | 1 |
| Cancer | Boolean | 0 | 0 | 1 |
| Cardiac Arrest | Boolean | 0 | 0 | 1 |
| Cardiomyopathy | Boolean | 0 | 0 | 1 |
| Chronic Heart Disease | Boolean | 0 | 0 | 1 |
| Chronic Liver | Boolean | 0 | 0 | 1 |
| Chronic Kidney Disease | Boolean | 0 | 0 | 1 |
| Coagulopathy | Boolean | 0 | 0 | 1 |
| Complication Lung Tx | Boolean | 0 | 0 | 1 |
| COVID | Boolean | 0 | 0 | 1 |
| Diabetes | Boolean | 0 | 0 | 1 |
| Haemorrhage | Boolean | 0 | 0 | 1 |
| Heart Tx Complication | Boolean | 0 | 0 | 1 |
| Hepatic Failure | Boolean | 0 | 0 | 1 |
| Hypertension | Boolean | 0 | 0 | 1 |
| Immunosuppressed | Boolean | 0 | 0 | 1 |
| Narcotics | Boolean | 0 | 0 | 1 |
| Neurological | Boolean | 0 | 0 | 1 |
| Obesity | Boolean | 0 | 0 | 1 |
| Pre-ECMO Cardiothorac Sx | Boolean | 0 | 0 | 1 |
| Pre-ECMO Lung Transplant | Boolean | 0 | 0 | 1 |
| Peripheral Vascular Disease | Boolean | 0 | 0 | 1 |
| Pregnancy | Boolean | 0 | 0 | 1 |
| Pulmonary Hypertension | Boolean | 0 | 0 | 1 |
| Post/periop | Boolean | 0 | 0 | 1 |
| Sepsis | Boolean | 0 | 0 | 1 |
| Thorax Disease/Pneumonitis | Boolean | 0 | 0 | 1 |
| Thromboembolic Disease | Boolean | 0 | 0 | 1 |
| Thrombosis | Boolean | 0 | 0 | 1 |
| Valvular Disease | Boolean | 0 | 0 | 1 |
| ARDS Risk Factors | | | | |
| RF Aspiration | Boolean | 0 | 0 | 1 |
| RF Bacterial Infection | Boolean | 0 | 0 | 1 |
| RF Burns | Boolean | 0 | 0 | 1 |
| RF Drowning | Boolean | 0 | 0 | 1 |
| RF Extrapulmonary infection/inflammation | Boolean | 0 | 0 | 1 |
| RF Infection, other | Boolean | 0 | 0 | 1 |
| RF Other | Boolean | 0 | 0 | 1 |
| RF Resp Neoplasm | Boolean | 0 | 0 | 1 |
| RF Transfusion | Boolean | 0 | 0 | 1 |
| RF Viral Infection | Boolean | 0 | 0 | 1 |
| RF Trauma | Boolean | 0 | 0 | 1 |
| Outcomes | | | | |
| Discharged Alive | Boolean | 0 | 0 | 1 |

## Supplemental Table 3

Threshold study exploring changes in precision and accuracy for the ECMO PAL model at different cutoff values to classify survival (0.5 value used in deployed model). AUC – Area under the receiver operating characteristic curve.

| **Threshold (≥)** | **Accuracy (%)** | **AUC** | **Survival Sensitivity (%)** | **Survival Precision (%)** | **Non-Survival Sensitivity (%)** | **Non-Survival Precision (%)** |
| --- | --- | --- | --- | --- | --- | --- |
| 0.1 | 70 | 0.78 | 96 | 69 | 22 | 77 |
| 0.2 | 71 | 0.78 | 93 | 71 | 32 | 72 |
| 0.3 | 72 | 0.78 | 90 | 73 | 41 | 69 |
| 0.4 | 73 | 0.78 | 87 | 75 | 49 | 67 |
| 0.5 | 73 | 0.78 | 81 | 77 | 57 | 63 |
| 0.6 | 71 | 0.78 | 77 | 80 | 66 | 59 |
| 0.7 | 69 | 0.78 | 65 | 84 | 77 | 75 |
| 0.8 | 64 | 0.78 | 51 | 88 | 87 | 49 |
| 0.9 | 54 | 0.78 | 32 | 92 | 95 | 44 |

## Supplemental Table 4

Survival outcome for the training and validation datasets.

| Dataset | Survived | Mortality |
| --- | --- | --- |
| Training (N = 35,182) | 59.4% | 40.6% |
| Temporal Validation (N = 4,318) | 64.4% | 35.6% |

## Supplemental Figure 3


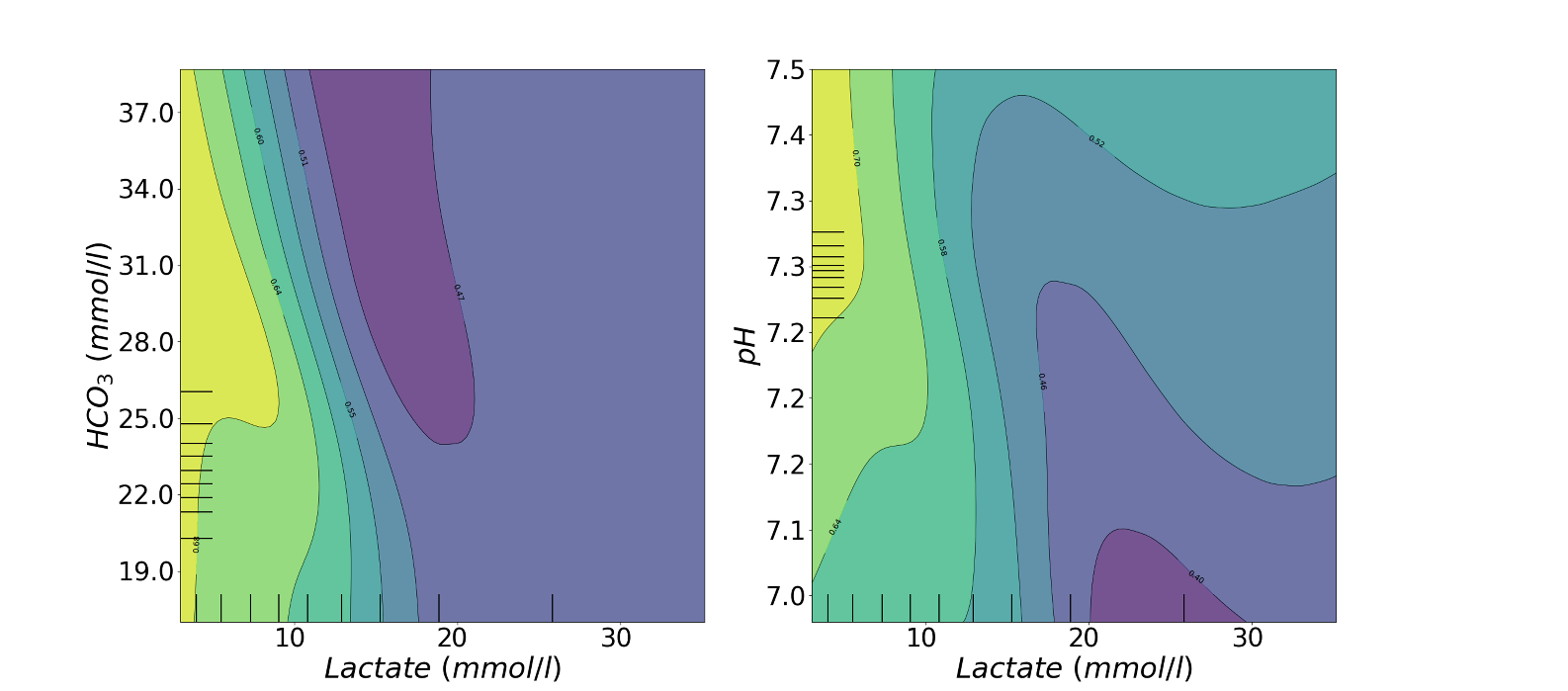


Figure 3: Partial dependence plots show the interaction between serum bicarbonate (HCO_3_) and pre-ECMO lactate (left) and pH and pre-ECMO lactate (right). Areas shaded in green are variable combinations which drive higher survival, while areas shaded in purple are variable combinations that drive hospital mortality. Black bars at the bottom denote decile boundaries.

## Supplemental Figure 4


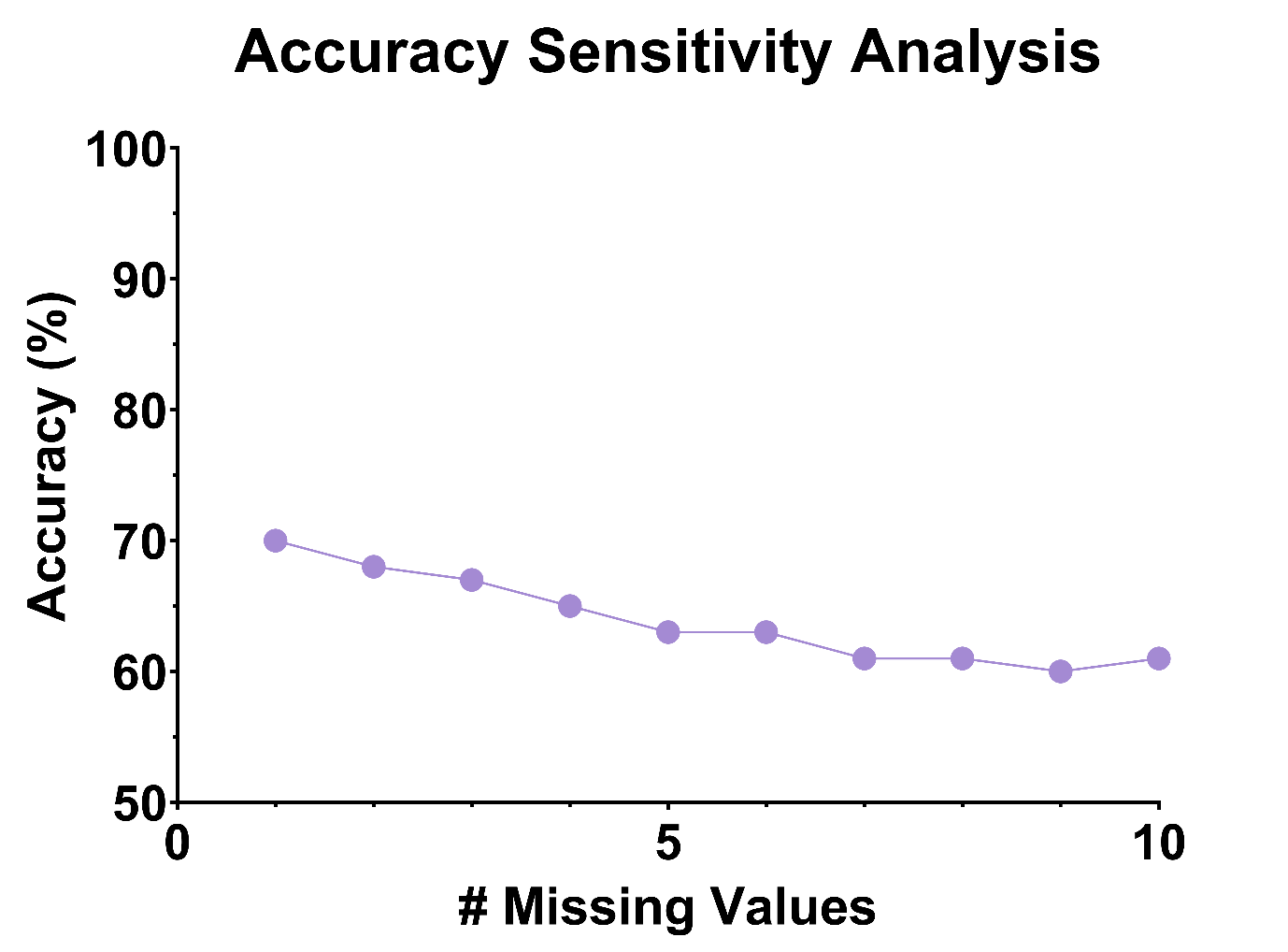


Figure 4: Sensitivity analysis showing the effect of missing data that has been median imputed on ECMO PAL: V-V accuracy.

## Supplemental Figure 5


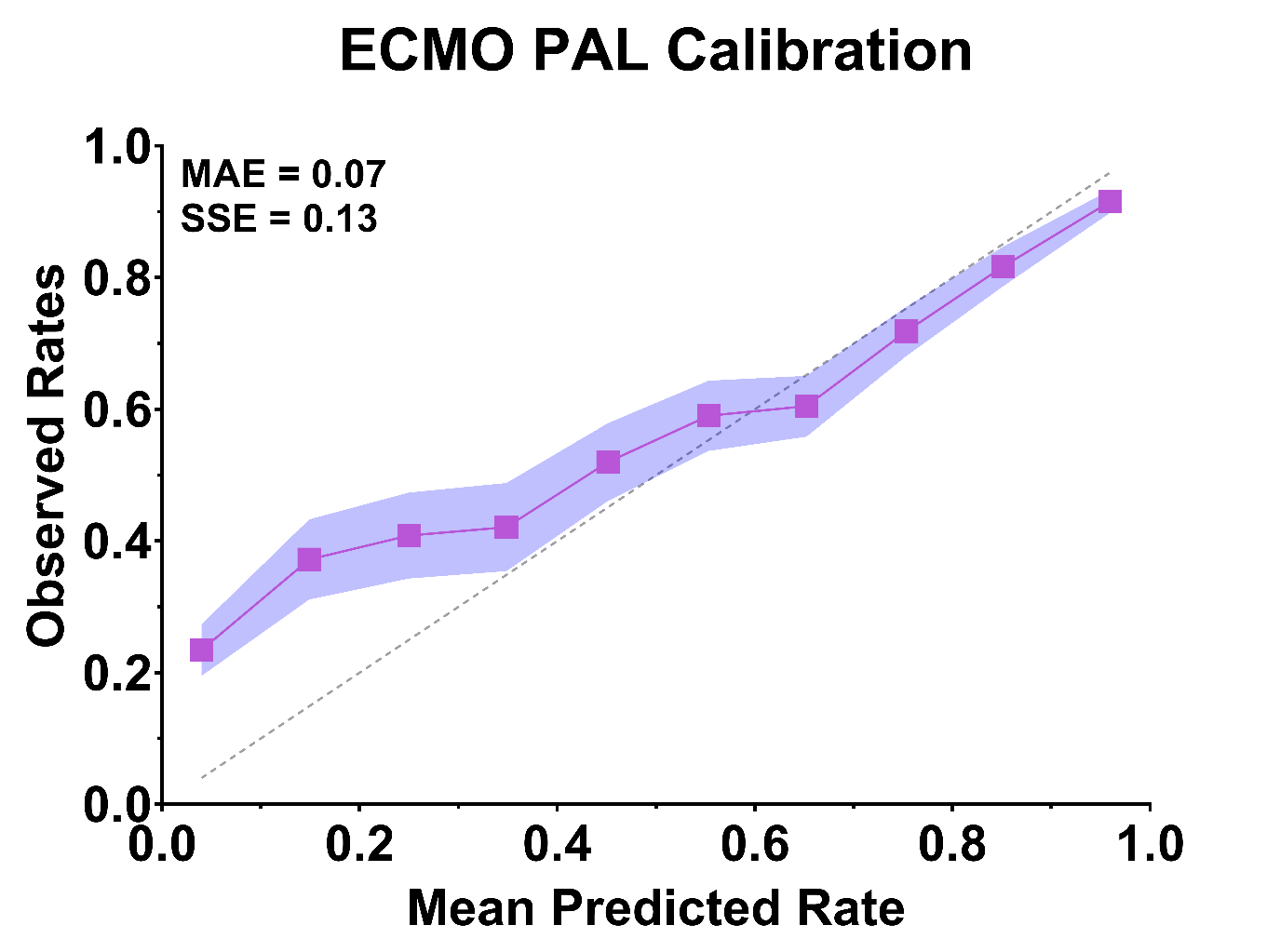


Figure 5: Calibration curve of ECMO PAL: V-V model. The dotted grey line represents perfect calibration. The shaded areas represent the upper and lower 95% confidence intervals.

## Supplemental Figure 6


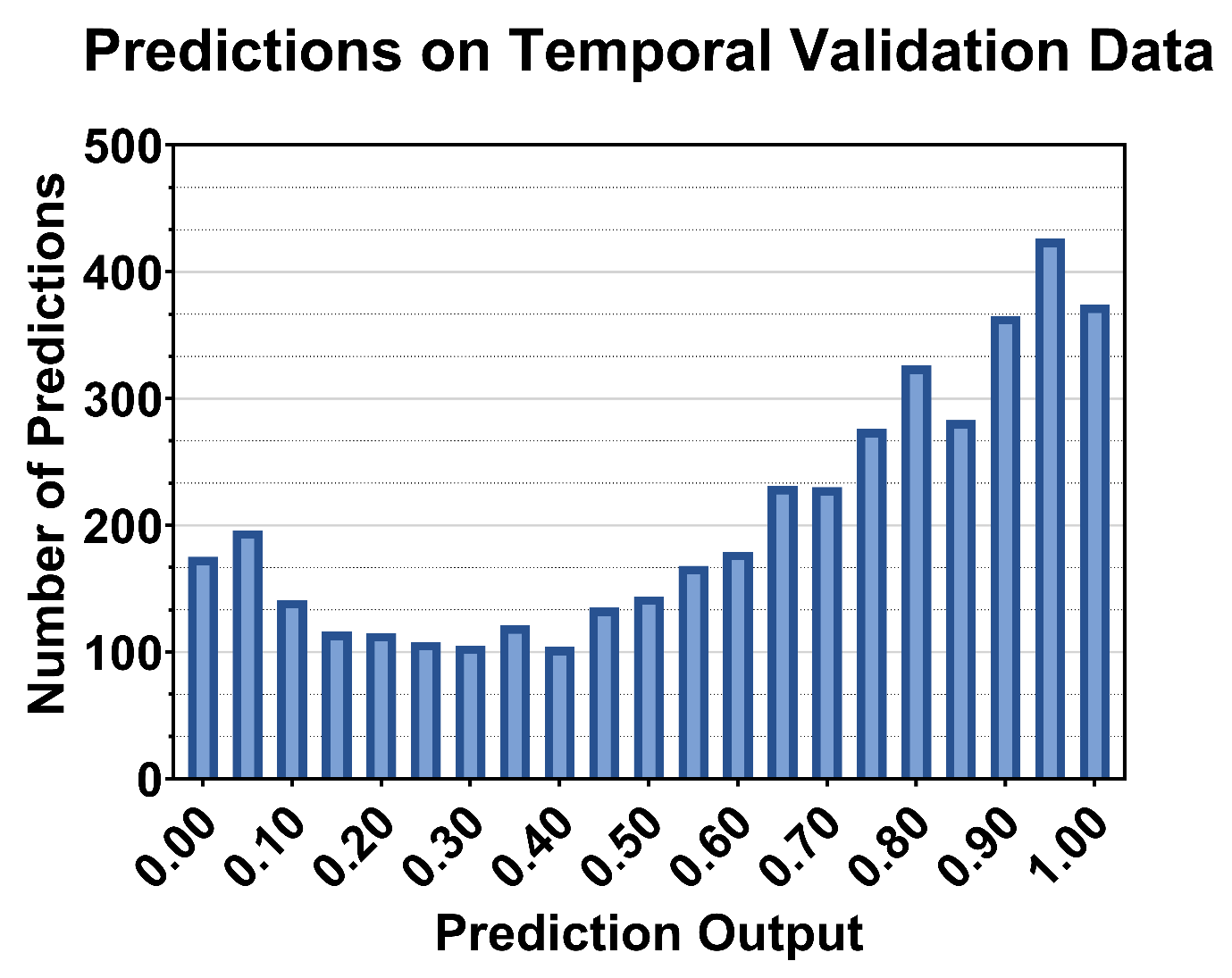


Figure 6: Histogram of model prediction outputs on the temporal validation dataset. The global survival of VV ECMO is 59%.

## Supplemental Table 4

Table 4: Sensitivity analysis comparing the inclusion of COVID-19 as a model variable. Analysis conducted by training the model with and without COVID as a variable and then making predictions on the validation data.

| **Metric** | **Without COVID** | **With COVID** |
| --- | --- | --- |
| **Training Fit Metrics** | | |
| *Training Accuracy (%)* | 79 | 79 |
| *Training AUC* | 0.79 | 0.87 |
| *Training Survival Sensitivity (%)* | 85 | 86 |
| *Training Survival Precision (%)* | 80 | 80 |
| *Training Mortality Sensitivity (%)* | 71 | 69 |
| *Training Mortality Precision (%)* | 77 | 77 |
| **Validation Metrics** | | |
| *Validation Accuracy (%)* | 73 | 70 |
| *Validation AUC (%)* | 0.78 | 0.72 |
| *Validation Survival Sensitivity (%)* | 81 | 82 |
| *Validation Survival Precision (%)* | 77 | 76 |
| *Validation Mortality Sensitivity (%)* | 57 | 52 |
| *Validation Mortality Precision (%)* | 63 | 61 |
